# Development of family and dietary habits questionnaires: the assessment of family processes, dietary habits and adolescents’ impulsiveness in Norwegian adolescents and their parents

**DOI:** 10.1186/s12966-014-0130-z

**Published:** 2014-10-15

**Authors:** Mona Bjelland, Solveig ES Hausken, Ester FC Sleddens, Lene F Andersen, Hanne C Lie, Arnstein Finset, Lea Maes, Elisabeth L Melbye, Kari Glavin, Merete W Hanssen-Bauer, Nanna Lien

**Affiliations:** Department of Nutrition, Faculty of Medicine, University of Oslo, P.O.Box 1046, Blindern, NO-0316 Oslo, Norway; Department of Health Promotion, Nutrition and Toxicology Research Institute Maastricht (NUTRIM), Maastricht University Medical Center+, Maastricht, The Netherlands; Department of Behavioural Sciences in Medicine, Faculty of Medicine, University of Oslo, Oslo, Norway; Public Health Department, Ghent University, Ghent, Belgium; University of Stavanger, UiS Business School, Stavanger, Norway; Department of Nursing, Faculty of Health Sciences, Oslo and Akershus University College of Applied Sciences, Oslo, Norway; Department of Nursing, Diakonova University College, Oslo, Norway

**Keywords:** Family, Adolescent, Diet, Impulsivity, Questionnaire, Validity, Reliability

## Abstract

**Background:**

There is a need for valid and comprehensive measures of parental influence on children’s energy balance-related behaviours (EBRB). Such measures should be based on a theoretical framework, acknowledging the dynamic and complex nature of interactions occurring within a family. The aim of the Family & Dietary habits (F&D) project was to develop a conceptual framework identifying important and changeable family processes influencing dietary behaviours of 13–15 year olds. A second aim was to develop valid and reliable questionnaires for adolescents and their parents (both mothers and fathers) measuring these processes.

**Methods:**

A stepwise approach was used; (1) preparation of scope and structure, (2) development of the F&D questionnaires, (3) the conducting of pilot studies and (4) the conducting of validation studies (assessing internal reliability, test-retest reliability and confirmatory factor analysis) using data from a cross-sectional study.

**Results:**

The conceptual framework includes psychosocial concepts such as family functioning, cohesion, conflicts, communication, work-family stress, parental practices and parental style. The physical characteristics of the home environment include accessibility and availability of different food items, while family meals are the sociocultural setting included. Individual characteristics measured are dietary intake (vegetables and sugar-sweetened beverages) and adolescents’ impulsivity. The F&D questionnaires developed were tested in a test-retest (54 adolescents and 44 of their parents) and in a cross-sectional survey including 440 adolescents (13–15 year olds), 242 mothers and 155 fathers. The samples appear to be relatively representative for Norwegian adolescents and parents. For adolescents, mothers and fathers, the test-retest reliability of the dietary intake, frequencies of (family) meals, work-family stress and communication variables was satisfactory (ICC: 0.53-0.99). Barratt Impulsiveness Scale-Brief (BIS-Brief) was included, assessing adolescent’s impulsivity. The internal reliability (Cronbach’s alphas: 0.77/0.82) and test-retest reliability values (ICC: 0.74/0.77) of BIS-Brief were good.

**Conclusions:**

The conceptual framework developed may be a useful tool in guiding measurement and assessment of the home food environment and family processes related to adolescents’ dietary habits, in particular and for EBRBs more generally. The results support the use of the F&D questionnaires as psychometrically sound tools to assess family characteristics and adolescent’s impulsivity.

**Electronic supplementary material:**

The online version of this article (doi:10.1186/s12966-014-0130-z) contains supplementary material, which is available to authorized users.

## Background

In a life-course perspective the home environment is the first to shape energy balance-related behaviours (EBRBs) such as dietary habits and physical activity [1,2]. Previous studies have indicated that a positive family system may be part of a process establishing and maintaining beneficial health behaviours through role-modelling, provision of healthy foods and support for engaging in healthy behaviours [3,4]. Earlier Norwegian studies have reported low vegetable intake and high energy intake from added sugar and sugar-sweetened beverages (SSB) in children and adolescents [5-9]. However, there seems to have been a recent decrease in SSB intake among Norwegian 11–13 year olds [10].

Several models and frameworks have been developed, which aim to explain how dietary habits of children and adolescents are related to the home food environment [1,3,11-13]. To our knowledge, however, none of these have applied an *ecological* framework to understand how processes within the family influence dietary habits in children and adolescents. An ecological perspective implies that behaviours or health outcomes result from the interaction between individual and environmental factors. In adolescence this includes the interplay between individual characteristics (such as gender and impulsivity), family processes (for instance family functioning, cohesion and conflicts) and context characteristics (such as family structure and sociocultural settings) [14]. How intrafamilial processes (e.g. inter-personal relationships like parent-parent and parent–child) and extrafamilial conditions (e.g. cross-level interactions such as conditions of parental work and socio economic status) affect families in fostering the healthy development of children needs to be explored [15]. However, little research has applied such socio-ecological frameworks to understand how parents influence children’s EBRBs [16].

Conceptualization of concepts related to the home food environment is needed [17,18]. Recently, based on the EnRG framework (Environmental Research framework for weight Gain prevention), Kremers et al. [19] stressed the need for “research that applies measures that have increased validity and comprehensiveness as well as theoretical frameworks that acknowledge the dynamic interplay of types and levels of parental influence on child EBRBs.” Additionally, the need for development and validation of methods of how to assess environmental influence on diet has been highlighted by Elinder and Jansson [20]. Kitzmann and Beech [21] have also emphasized the importance of exploring a broader focus on the family context, such as general parenting and family functioning, in the promotion of healthy eating and regulation of unhealthy eating among adolescents.

More recently, it has been acknowledged that few studies have combined personality constructs with environmental factors in the prediction of EBRBs [19,22]. In the four factor model of temperament described by Buss and Plomin [23], impulsivity is included along with emotionality, activity and sociability. Impulsiveness can be defined as “a predisposition toward rapid, unplanned reactions to internal or external stimuli without regard to the negative consequences of these reactions to the impulsive individuals or to others” [24]. Sleddens et al. [25] found that parental monitoring of children’s snacking was moderated by the impulsivity of the child. To specify, for children with high scores on effortful control and extraversion, monitoring was related to less snacking. Therefore, a child’s impulsivity may have important consequences for the development, maintenance and treatment of obesity [26].

In sum, an ecological framework including individual characteristics, intrafamilial processes and extrafamilial conditions, combined with instruments developed to assess these aspects within family units, may be a valuable contribution to explore the role of families in fostering healthy development of children and adolescents. The overall aims of the Family & Dietary habits project (the F&D project) were therefore to develop a conceptual framework, applied to understand the family influences upon dietary habits in adolescents. Furthermore, to develop valid and reliable questionnaires measuring these important and changeable family processes potentially influencing dietary behaviours for 13–15 year olds and their parents (both mothers and fathers). It would be too demanding to test the questionnaires on all aspects of adolescents’ dietary habits, so two behaviours were selected; vegetable intake representing a healthy eating behaviour to be promoted, and consumption of SSB representing an unhealthy eating behaviour to be regulated. These behaviours were also chosen because they are regarded as important contributors to child health and the prevention of obesity and related chronic diseases [27-29].

The current article presents 1) the development of the conceptual framework and the questionnaires, and 2) the individual characteristics, context and extrafamilial conditions of the study sample to be used for further reliability and validation studies. Measures characterizing intrafamilial processes are not included, except for communication which was reported by adolescents only. The psychometric properties of the Barratt Impulsiveness Scale-Brief (BIS-Brief) in Norwegian 13–15 year olds and their parents for assessing adolescents’ impulsivity are also included, as a personality characteristic potentially moderating family processes.

## Method

### Framework and questionnaire development

**Figure 1:**
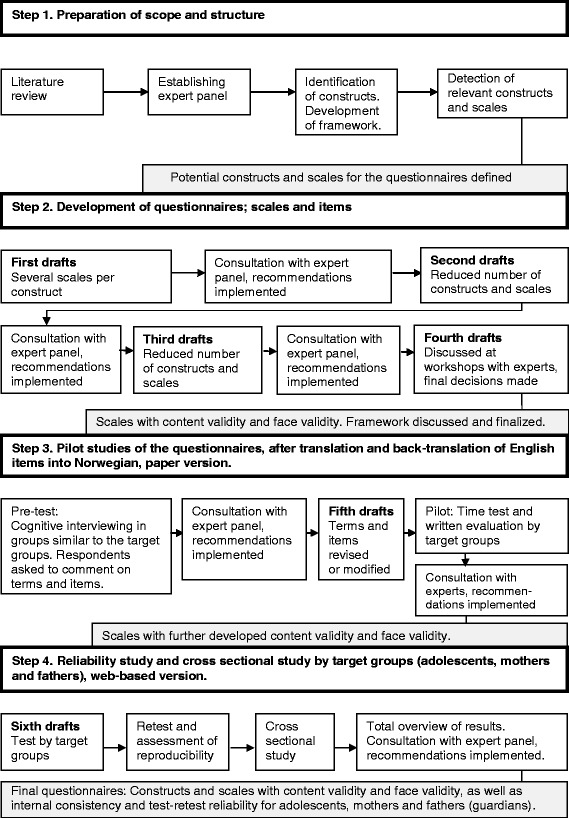
**The process applied in the development of questionnaires in the Family & Dietary habits project.**

The process for developing the framework and the F&D questionnaires is summarized in Figure 1 and described below in four steps.

#### Step 1: Preparation of scope and structure

The initial step involved the identification of relevant concepts and the development of a conceptual framework, based an assessment of relevant literature and in collaboration with an expert group. The expert group included five professors, four postdoctoral researchers and one lecturer in different scientific fields related to family processes and dietary habits (nutrition, behavioural sciences, nursing, clinical nutrition, public health, psychology and health promotion).

### Definitions

The following definition of a family was agreed upon: A family is a group consisting of one or two parent(s)/guardian(s) and one or more children, within the same household. The home food environment is considered to include events (meals/family meals), objects (household availability of food) and social interactions (family processes) experienced by children and parents in the family context, based on previous definitions [30,31]. The family processes in this project include psychosocial characteristics as well as more physical characteristics such as accessibility in accordance with Smith et al. [32].

### Dietary behaviours

Intake of SSB was assessed by frequency (six categories, from never/seldom to every weekday) and amount (in glasses, four categories: from 1 glass to 4 glasses or more) for weekdays and by amount for weekends (in glasses, eight categories: from never/seldom to 7 glasses or more) [33]. Vegetable intake was assessed by eight pre-coded frequencies from never to three times or more per day for two questions on raw and cooked vegetables [33].

### Target groups

We hypothesized that the age of the child is one important characteristic, including 13–15 year olds and their parents in our study. In the Norwegian setting, adolescents enter secondary school at the age of 13. Starting secondary school coincides with several shifts in physical, social and cognitive functioning, making this a highly relevant time for studying family processes [34]. After the age of 15 years adolescents begin to experience greater autonomy in decision making [35].

Results from previous studies suggest that the opposite sex parent may play a unique role in influencing adolescent health behaviours, and that there are differences in maternal and paternal parenting practices/styles [36-38]. Gender-specific results are of importance in order to identify underlying causal mechanisms, and therefore the expert group wanted adolescents to report separately for mothers and fathers for relevant constructs related to parenting practices/style.

#### Step 2: Development of questionnaires; scales and items

Drafts of the F&D questionnaires were made based on the literature and the evaluations given by the expert group. During correspondence through e-mail and two workshops the expert group assessed the content and face validity of the instruments [39].

#### Step 3: Pilot studies of the questionnaires, after translation and back-translation of English items into Norwegian, paper version

A translation and back-translation of scales/questionnaires available in English were conducted by fluent speakers of the English and Norwegian languages, and a small time-test (paper-versions, 5 adolescents and 2 adults) was conducted. Items were removed or adapted to a Norwegian setting after consulting members of the expert group. A pre-test was conducted in three separate groups, each consisting of three mothers, three fathers and five 13-year-olds, two boys and three girls. Cognitive interviewing was conducted to ensure that adolescents and parents understood the instructions, items and response scales. Moreover, the pre-test consisted of a discussion of whether respondents understood particular words/phrases as intended, and discussion of items identified as complex. Members of the expert group were consulted regarding removal or adaption of items based on the pre-test.

After the pre-test, a pilot study was conducted. It included a time test and a written evaluation of paper-versions of the full F&D questionnaires by 17 adolescents (13 years of age; 9 girls, 8 boys) and 14 parents (9 mothers, 5 fathers). The participants were asked to report if there were any questions or statements perceived as particularly unclear/difficult to answer, if any questions or statements should be moved to another part of the questionnaire, and if anything important or relevant was missing. When time testing the paper version of the F&D questionnaires, the adolescent questionnaire was completed in about 25–45 minutes (average time: 36 minutes) (n= 17), and the parental questionnaires in about 20–45 minutes (average time: mothers 30 minutes, fathers 23 minutes) (n= 14).

#### Step 4: Reliability study and cross-sectional study in the target groups (adolescents, mothers and fathers), web-based version

All participants were recruited through a convenience sample of five secondary schools – one in the county of Oslo and four in the neighbouring county of Akershus. In total, 1136 adolescents were invited to participate in the cross-sectional study, of which 440 adolescents (13–15 year olds, 39%) and 397 of their parents (242 mothers (55%) and 155 fathers (35%)) participated. Of these, 204 were invited to participate in a test-retest of the web-based versions of the full F&D questionnaires, and 54 adolescents (26%) and 44 of their parents (33 mothers and 11 fathers) participated. The test and retest were conducted 10–14 days apart. The participants were rewarded by providing a small money contribution for the school class (e.g. for school trips). Informed parental consent was obtained from all participants. The web-based F&D questionnaires were mainly comprised of questions with pre-coded answer categories and the data collections took place at school. Information about how to access the web-based parental questionnaires, one for each parent, was brought home by the adolescent. Most parents provided their e-mail address on the consent form, and thus the information was also sent by e-mail. One e-mail reminder was sent to parents. The Norwegian Social Science Data Services has approved the study and The Regional Committees for Medical and Health Research Ethics has been informed, but no approval was needed.

### Data analysis

The characteristics of the cross-sectional samples are presented as proportions (demographic variables), means and standard deviations (SD) (behavioural, meals, stress and communication variables). Intra-class correlation coefficient analyses (ICC, a two-way random effects single measure) were used to assess the test-retest reliability. The ICCs were classified as follows: “excellent” (≥0.81), “good” (0.61 - 0.80), “moderate” (0.41 - 0.60), ‘poor’ (≤0.40) [40,41]. Because the calculation of the ICC depends on the existence of the variability in answers [42], we also calculated percentage agreement for the cases with ICC below 0.5, with criteria established as “excellent” (90% - 100%), “good” (75% - 89%), “moderate” (60%-74%), or “poor” (<60%) [40].

For the BIS-Brief, Corrected Item-Total Correlation (CITC) and Cronbach’s alpha were used to assess the internal reliability of the construct. CITC >0.30 were considered good, and<0.15 were considered unreliable since they would indicate lack of homogeneity of the items within a scale [43]. Cronbach’s alpha was classified as >0.70= “acceptable” and >0.80= “preferable” [44]. Confirmatory factor analysis (CFA) was used to test whether the impulsivity data using the BIS-Brief would fit a hypothesized measurement model based on theory and previous analytic research. Acceptable CFA model fits were Root Mean Square Error of Approximation (RMSEA)<0.06-0.08 and Comparative Fit Index (CFI) ≥0.95 [45].

The descriptive and reliability analyses were performed using IBM® PASW® Statistics, version 20.0 (IBM Corp., Somers, New York, USA). The IBM® SPSS® Amos (IBM Corp., Somers, New York, USA) was used for conducting the CFA.

## Results

### Ecological framework

**Figure 2:**
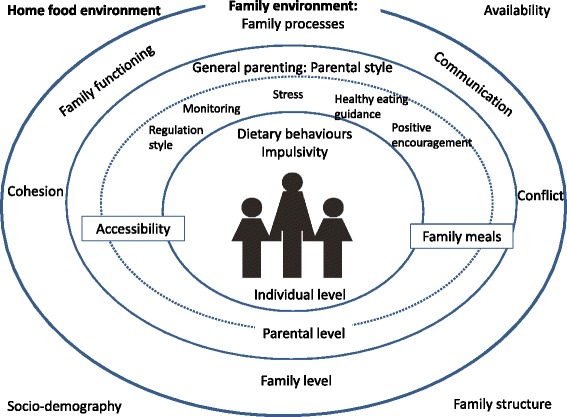
**The ecological framework developed in the Family & Dietary habits project.**

Based on models/frameworks, literature, inputs from the expert group, and the definitions and aims presented above, an ecological framework was developed describing the different levels and constructs included in the F&D questionnaires (Figure 2). The rationale for including these constructs and levels is described below.

Recent literature points to the importance of investigating more general measures of family functioning as contexts that can enhance or limit the effectiveness of family-based interventions [21,38]. We therefore included measures of family functioning, cohesion, conflict and communication. Two important factors at the parental level are parenting style (a set of attitudes that create a certain emotional climate) and parenting practices (specific behaviours defined by specific socialization goals) [21,46]. General parenting determines the context of behaviour specific parenting [46], while specific examples of parenting practices include pressure to eat, restriction, monitoring of the child’s food intake, and the use of rewards for food consumption [47].

Another important factor is family meals. The frequency of shared family meals is significantly related to a healthier dietary pattern in children and adolescents [48,49]. Moreover, the presence of at least one parent at meals is reported to be positively associated with a higher consumption of fruit and vegetables [50]. The relevance of the physical home environment (e.g. availability and accessibility) has been confirmed in several reviews [51-53]. Availability concerns whether foods of interest are present in an environment, while accessibility concerns whether these foods are available in a form, location, and time that facilitates their consumption [54]. Impulsivity is positively associated with several facets of unhealthy eating [55] and weight [56]. Moreover, people who are impulsive tend to be worse at inhibiting and controlling their responses to signals, one of which is the tendency to eat [55]. Adolescent’s impulsivity, assessed by BIS-Brief [57], was included at the individual level as a potential moderator of family processes that may influence dietary behaviours [55,56]. The BIS-Brief is a short one-dimensional version of the original BIS-11 version [46].

### The F&D questionnaires

The constructs included in the F&D questionnaires are presented in Table 1 by the order as in the questionnaires, including references to the original scales. Most of the scales/questionnaires included were from previous studies [25,33,57-67] with the following adaptations for the F&D questionnaires.

**Table 1:** **The constructs included in the questionnaires within the Family & Dietary habits project**

**Constructs in the questionnaires**	**Number of items**		**Reference**
	**Adolescents**	**Parents**	
Socio-demography	10	11 (2 routed)	Lien et al. [33]
Meals; Breakfast, dinner and family meals	6		Lien et al. [33]
Dietary intake; Vegetables and SSB (amount)	38 (2 vegetables, 36 SSB:12 frequency/12 amount week days, 12 amount weekend)		Lien et al. [33]
Accessibility; Vegetables and SSB	25 (16 accessibility, 9 accessibility/availability)		Lien et al. [33], Sirard et al. [58]
Availability*; Vegetables and SSB	-	24	Lien et al. [33], Hearn et al. [65]
Family meals (priority, atmosphere, structure)	9		Fulkerson et al. [59]
Activities during meals (breakfast, dinner)	8		Fulkerson et al. [59]
Communication*	3	-	Tabak et. al [60]
Family assessment devise (general functioning)	12		Ridenour et al. [61]
Family environment scale (cohesion/conflict)	17		Moos and Moos [67]
Work-family stress*	-	3	Bauer et al. [64]
Positive encouragement: Vegetables	10 (5 for mother and 5 for father)	10 (5 routed)	Dave et al. [62]
Comprehensive Feeding Practices Questionnaire (healthy eating guidance/monitoring)	26 (13 for mother and 13 for father)	17 (2 routed)	Haszard et al. [63]
Parental style of regulation: SSB, sweets, snacks	30 (15 for mother and 15 for father)	15	Vansteenkiste et al. [66]
Comprehensive General Parenting Questionnaire (nurturance, structure, behavioural control, overprotection, coercive control)	98 (49 for mother and 49 for father)	49	Sleddens et al. [70]
Brief Barratt Impulsiveness Scale	8		Steinberg et al. [57]
Weight and height	2		Lien et al. [33]

*Adolescents or parents only. Routed: items/questions following specific answer categories.

SSB: Sugar sweetened beverages.

The original Comprehensive Feeding Practices Questionnaire (CFPQ) [68] was developed for parents of children aged 2–8 years. It consists of 49 items representing 12 dimensions (subscales), each including 3–8 items. Initial testing of the CFPQ with parents of older children in a Norwegian setting indicated that the instrument, with some small modifications, was a valid tool for measuring multiple parental feeding practices with parents of 10–12 year olds [69]. However, Haszard et al. [63] performed a CFA on the CFPQ in a large, diverse sample (n=1013) of New Zealand parents of children aged 4–8 years. The results showed that the original twelve-factor model (49 items) was not a good fit and that several factors were strongly inter-correlated. A subsequent CFA yielded five scales of interest (32 items): healthy eating guidance (9 items), monitoring (4 items), parent pressure (7 items), restriction (8 items) and child control (4 items). Two of the scales were included in our questionnaires (healthy eating guidance and monitoring).

Additionally, Dave et al. [62] have developed a measure of parent-reported social support (instrumental and emotional) for children’s fruit and vegetable intake, identifying four subscales; positive encouragement, negative role modelling, discouragement to eat fruit and vegetables and reinforcement. We modified the positive encouragement subscale to cover intake of vegetables only [62]. Moreover, when parents reported that they never or seldom encourage their child, a follow-up question with three response options was given; not necessary (due to high intake), not important to me (attitude) or my child is old enough to decide him-/herself (child’s autonomy). For healthy eating guidance, monitoring and positive encouragement, both the adolescent perspective (reporting both for the mother and the father) and the parental perspective (from both mothers and fathers) were included.

The parental feeding practices included in our study are practices that could be recommended to promote healthy dietary behaviours; meaning healthy eating guidance, monitoring and positive encouragement. Because we had to limit the number of questions/statements in the questionnaires, other parenting practices were excluded in the F&D project.

The assessment of parental style of regulation was based on work by Vansteenkiste et al. [66], who developed a measure for parental autonomy-supportive and controlling style in the domain of eating regulation. A modified version assessing an autonomy-supportive, covertly and overtly controlling style in regulation of SSB, sweets and fatty/salty snacks was used in our study. Statements dealing with the way the mother and father regulate intake of SSB, sweets and fatty/salty snacks were presented for the adolescents and the parents. The participants were asked to indicate for each of the statements to what extent it applied to the mother/father on a 5-point scale.

The Comprehensive General Parenting Questionnaire (CGPQ) was systematically developed by Sleddens et al. [70]. First, an item bank of existing parenting measures was created assessing five key parenting constructs: nurturance, structure, behavioural control, overprotection and coercive control. Then caregivers of 5–13 year olds in the Netherlands, Belgium and United States completed an online survey. Factor analyses and Item-Response Modelling techniques were used to assess the underlying parenting constructs and for item reduction. After adding additional questions for better coverage of some sub-factors, this resulted in an 85-item questionnaire. We included a 49-item version of the CGPQ, modified from the 85-item CGPQ by the developers [70], assessing the adolescent perspective (reporting both for the mother and the father) and the parental perspective (from both mothers and fathers). Adolescents and parents were asked to respond to statements about general parenting using a 5-point Likert scale ranging from 1 (strongly agree) to 5 (strongly disagree).

Finally, the Family Environment Scale is composed of 10 subscales that measure actual, preferred, and expected family social environments [67]. To limit the number of questions/statements in the questionnaires, we only included the subscales cohesion and conflict, based on recommendations from the expert group. The item “Family members sometimes hit each other” from the original conflict scale was removed because hitting children is forbidden by law in Norway. This was the only item removed from an original scale included in the questionnaires developed as part of the F&D project.

### Study sample characteristics

Characteristics of the cross-sectional study sample and the test-retest sample (adolescents and parents) are presented in Tables 2 and 3. The adolescents were on average 14.3 (0.6) years and the genders were equally divided. Most of the adolescents lived together with both parents, while 15.7% lived with only/mostly the mother or father. In total 66.2% of the adolescents had parents with higher education (≥13 years), and 9.1% were not ethnic Norwegians (meaning that both parents were born in another country than Norway) [71]. The proportions of girls and highly educated parents were higher in the test-retest sample than in the cross-sectional sample. The distribution of ethnicity and average age was about the same for the mothers and the fathers, while there were more fathers of boys participating compared to fathers of girls. Most of the parents participating lived with the child the whole time while there were a higher proportion of fathers living together with the child’s mother than vice versa. The proportion of fathers working full-time was higher compared to mothers, while a higher proportion of mothers worked part-time (Table 2). There was a higher proportion of parents of girls in the test-retest sample than in the cross-sectional sample. For both adolescents and parents the consumption of SSB (soft drink and cordials with sugar) was low (mean daily intake on week days was ≤1.5 dl/day), and the intake of vegetables was ≤10 times per week (Table 3). The means of breakfast and dinner were ≥6 times per week, while the means of family meal frequencies were lower. The parental perceived work-family stress was low (close to 3 on a scale from 1 (strongly agree) to 4 (strongly disagree) in perceived stress). There were 131 dyads (29.8%) and 128 triads (29.1%) in the cross-sectional study sample.

**Table 2:** **Characteristics (demographic) for the adolescents and parents in the study sample**

	**Adolescents**		
	**n** ^**†**^ **=440**	**n#=54**	
**Age** (mean (SD))	14.3 (0.6)	13.9 (0.3)	
**Gender**			
Boys (%)	47.7	40.7	
Girls (%)	52.3	59.3	
**Live together with**			
Mother and father (%)	68.7	71.7	
Only/mostly with mother (%)	13.0	17.0	
Only/mostly with father (%)	2.7	1.9	
Equal time with mother/father (%)	10.0	9.7	
Mother and her new partner (%)	4.1	0	
Father and his new partner (%)	0.9	0	
Other adults (%)	0.5	0	
Ethnicity*			
Norwegian (%)	90.9	88.7	
Other ethnicity (%)	9.1	11.3	
**Parental educational level**			
< 12 years (%)	33.8	9.3	
13-16 years (%)	39.3	37.0	
> 16 years (%)	26.9	53.7	
	**Mothers**	**Fathers**	**Parents**
	**n** ^**†**^ **=242**	**n** ^**†**^ **=155**	**n#=44**
**Age** (mean (SD))	44.2 (4.5)	45.8 (5.5)	46.2 (4.4)
**Gender of child**			
Boys (%)	49.2	56.8	40.9
Girls (%)	50.8	43.2	59.1
**Ethnicity**			
Born in Norway (%)	90.0	87.1	84.1
Born in other country (%)	10.0	12.9	15.9
**Live together with the child (%)**			
The whole time	87.5	89.7	93.2
More than 50% of the time	7.5	2.6	2.3
Half the time (50% of the time)	4.6	5.2	4.5
Less than 50% of the time	0.4	1.9	0
Seldom	0	0.6	0
**Live together with the child’s mother/father**			
Yes (%)	75.2	88.3	83.7
Live alone (%)	15.3	5.8	14.0
Live with adult who is not child’s parent (%)	9.5	5.8	2.3
**Employment status**			
Working full-time (%)	68.5	94.2	77.3
Working part-time (%)	22.4	1.9	13.6
Not working for pay (%)	9.1	3.9	9.1

^†^Adolescents; n=417-440 (# test-retest sample; n=53-54), mothers; n=240-242, fathers; n= 154-155 (# test-retest sample; 43–44) *Other ethnicity: Both parents born in other country than Norway.

**Table 3:** **Characteristics and intraclass correlation coefficients for the dietary habits, meal frequencies, stress and communication**

	**Adolescents**			**Mothers**		**Fathers**		**Parents**
	**n** ^**†**^ **=440**		**n** ^**†**^ **=54**	**n** ^**†**^ **=242**		**n** ^**†**^ **=155**		**n** ^**†**^ **= ** **44**
**Dietary intake**	**Mean**	**SD**	**ICC**	**Mean**	**SD**	**Mean**	**SD**	**ICC**
Soft drink, dl/day, week	0.6	(1.0)	0.58	0.1	(0.7)	0.5	(0.9)	0.88
Soft drink, dl/day, weekend	2.0	(1.7)	0.78	0.7	(1.1)	1.0	(1.5)	0.92
Cordials, dl/day, week	0.9	(1.4)	0.53	0.2	(0.5)	0.4	(1.0)	0.89
Cordials, dl/day, weekend	0.7	(1.0)	0.33	0.2	(0.6)	0.4	(0.9)	0.74
Raw vegetables, times/week	5.6	(4.9)	0.63	6.3	(4.7)	4.7	(3.6)	0.82
Cooked vegetables, times/week	3.9	(2.6)	0.60	3.7	(2.0)	3.9	(1.9)	0.66
Total vegetables, times/week	9.5	(6.4)	0.69	10.0	(5.7)	8.6	(4.7)	0.80
**Meal frequencies**	**Mean**	**SD**	**ICC**	**Mean**	**SD**	**Mean**	**SD**	**ICC**
Breakfast, times/week	6.0	(1.9)	0.91	6.5	(1.5)	6.1	(1.9)	0.99
Dinner, times/week	6.8	(0.7)	0.49	6.8	(0.6)	6.7	(0.7)	0.14
Family meals; breakfast, times/week	4.5	(2.6)	0.73	4.9	(2.3)	4.0	(2.4)	0.91
Family meals; dinner, times/week	6.3	(1.3)	0.21	6.1	(1.3)	5.6	(1.4)	0.84
**Work-family stress** ^**#**^ **- reported by parents only**	**Mean**	**SD**	**ICC**	**Mean**	**SD**	**Mean**	**SD**	**ICC**
Because of the requirements of my job; − I miss out on home or family activities that I would prefer to participant in.	-	-	-	3.2	(0.9)	2.8	(0.9)	0.77
- my family time is less enjoyable or more pressured	-	-	-	3.3	(0.7)	3.1	(0.7)	0.71
Working leaves me with too little time or energy to be the kind of parent I want to be	-	-	-	3.1	(0.8)	2.9	(0.8)	0.31
Communication* - reported by adolescents only	**Mean**	**SD**	**ICC**	**Mean**	**SD**	**Mean**	**SD**	**ICC**
Your mother	1.7	(0.8)	0.69	-	-	-	-	-
Your father	2.1	(0.9)	0.72	-	-	-	-	-
Your sibling(s)	2.3	(1.0)	0.65	-	-	-	-	-

^†^n vary slightly; Adolescents n= 401–440, mothers: n=217-242, fathers: n=147-155. Intraclass correlation coefficient, ICC: Adolescents n= 50-54, parents: n= 39-44.

^**#**^Strongly agree (1) to strongly disagree (4), mothers; n=217-218, fathers; n=147: not answered by those who do not have a paid job.

*Very easy (1) to very difficult (4). Those who do not have or do not see this person(s) are excluded.

### Measures

For most of the items measuring intakes, meal frequency, work-family stress and communication the test–retest reliability was good (ICC >0.61) to excellent (ICC >0.81) (Table 3). For the items with an ICC ≤0.50 the exact/percent agreement was calculated, and ranged from moderate to excellent. For the adolescents the exact agreement for dinner was 96%, for family dinners 59%, for intake of cordials on week days 63% and for cordials intake on weekend days 62%. For adults the exact agreement for dinner was 84% and for work-family stress (“Working leaves me with too little time or energy to be the kind of parent I want to be”) it was 64% (data not shown).

The mean for adolescent self-rated impulsivity (Additional file 1) was 2.00 (SD 0.49) on a scale ranging from rarely/never (1) to almost always/always (4). The means for the parents’ report were 1.93 (SD 0.47) for mothers and 1.98 (SD 0.47) for fathers, respectively. For all three groups (adolescents, mothers, and fathers) the values of CITC were good (>0.30). The Cronbach’s alpha was considered acceptable for adolescents and parents (>0.70). The test–retest reliability of BIS-Brief for the three groups was good to excellent (ICC >0.61).

The factor loadings for adolescents, mothers and fathers in the analyses of the BIS-Brief were between 0.38 and 0.79. The fit indices for adolescents and fathers were not ideal, but acceptable. The model for mothers fitted the data best with the fit indices approaching acceptable CFA model fits (Additional file 2).

## Discussion

As part of the F&D project, questionnaires for adolescents and their parents (both mothers and fathers) have been developed based on a conceptual framework. The F&D questionnaires assess family processes that potentially influence dietary behaviours (intake of vegetables and SSB) in 13–15 year olds. The development process consisted of four steps; (1) preparation of scope and structure, (2) development of the F&D questionnaires, (3) conducting pilot studies and (4) conducting validation studies using data from a cross-sectional study. As a potential moderator of family processes influencing dietary behaviours, the adolescents’ impulsivity was included in our conceptual framework. The study samples appear to be relatively representative for Norwegian adolescents and parents. The test-retest reliability of the dietary intake, (family) meals, work-family stress and communication variables was satisfactory, and the internal reliability and test-retest reliability values of BIS-Brief were good.

### Framework and questionnaire development

The conceptual framework developed as part of the F&D project builds on the model of home food environment pertaining to childhood obesity by Rosenkranz and Dzewaltowski [1] and the EnRG framework by Kremers et al. [22], but is presented as an ecological framework [14,15]. The ecological framework allows researchers, parents and policy makers to conceptualize the home food environment and conditions that influence adolescents’ food choices within this environment. Furthermore, it illustrates the reciprocity among levels and the interrelationships among processes within the family. Finally, the framework can be applied to first understand the causes of adolescents’ dietary habits (i.e. determinant analysis) and then to develop strategic responses that bring about changes in these determinants. The F&D questionnaires developed were tested in a larger survey including multiple existing scales and questionnaires assessing individual characteristics, intrafamilial processes and extrafamilial conditions. Results from this assessment are currently being prepared for publication. The F&D project is unique in its attempt to incorporate several of these concepts into a comprehensive framework to disentangle the complex mechanisms of family influences on adolescents’ dietary habits.

### The study sample

Based on the characteristics measured in our study, the adolescents in the cross-sectional study seem to be relatively representative for Norwegian adolescents. When comparing with the national representative 13- and 15 year olds in the Norwegian sample of the 2009/2010 HBSC-study [72], the proportions of adolescents reporting living with both parents/single parent and eating breakfast every (week)day is about the same as in the F&D sample. The proportions of adolescents in the F&D sample who found it very easy/easy to talk to their mother or father about things that really bother them is somewhat higher compared to the HBSC sample, as is the adolescents’ daily intake of vegetables. This might be related to the parental education level as the level within the F&D sample (66% having ≥13 years of education) is higher than for a national representative sample of adults aged 40–49 (37% having ≥13 years of education) [73]. In general, immigrants account for about 12% of the Norwegian population, while the proportion of immigrants in the recruitment area for the F&D-project is higher (16-17%) [74,75]. However, the proportion of immigrants in the F&D sample of adolescents and adults is closer to the national level (9-13%). The proportion of adults in the F&D sample working full time is higher than for Norwegian adults at a national level, which may be related to the high educational level [76]. Work-family stress reported in the F&D sample is comparable to the work-to-family conflict level reported in another Norwegian study [77]. Taken together, the sample of adolescents in the F&D sample have higher educated parents compared to the general population, but otherwise the F&D samples seem relatively representative for Norwegian adolescents and parents.

The test-retest reliability (ICCs) for the adolescents’ intake of raw and cooked vegetables is comparable to those found for 11 year olds in the same geographic area in 2007 [78]. The ICCs for soft drink consumption were higher, while the reliability was lower for the assessment of cordial consumption, compared to the previous study [78]. The parental ICCs for beverage intakes were a bit lower compared to the mothers and fathers of the 11 year olds, while the reliability of the measures related to intake of vegetables was higher [78]. For both adolescents and parents the ICCs in our study were higher for the frequencies of breakfast and family breakfast compared to the cross European ENERGY study, including Norwegian 10–12 year olds and their parents [40,79]. The ENERGY study did not include questions about dinner. The ICCs for the work-family stress items are comparable to the test-retest reliability reported by Bauer et al. [64]. No reliability results are available for the measure of communication, but according to Tabak et al. [60] this measure has been “used and validated in numerous national and international studies as a good measure of the respondent’s relationship with each of their parents”. This is in accordance with the results from our study, showing good test–retest reliability.

Several Norwegian studies have been published related to impulsivity and diagnosis/ conditions like ADHD and self-harm. However, only one study was identified assessing impulsivity in a representative group of Norwegian adolescents (aged 14–17 years) and adolescents from six other countries [80]. The mean score for adolescents in all the seven countries indicated that they “sometimes” were impulsive, which is comparable to our mean which equals “occasionally”.

No other studies presenting test-retest reliability or conducting CFA for BIS-Brief in adolescents have been identified. Two studies report results from CFA of the full BIS 11-version in Chinese [81] and Italian [82] adolescents aged 13–19 years. The CFI [45] is one indicator of model fit which can be used to compare results across studies. For the adolescents in our study CFI=0.73, which was close to the results in the Chinese study (CFI=0.77/0.78), and higher than the CFI for the one-dimensional model in the Italian study (CFI=0.48).

### Strengths and limitations

The strengths of the questionnaires developed through the F&D project is the conceptual framework that acknowledges the dynamic interplay of types and levels of the home food environment and parental influence, derived from previous solid models and frameworks. Moreover, the F&D questionnaires include the same questions for both adolescents’, mothers and fathers, giving the unique opportunity to explore associations between family processes and dietary behaviours, bidirectional relationships and gender dyads. The main limitations of the questionnaires developed are the length and the assessment of only two dietary behaviours. To be able to assess relevant family processes, several scales/questionnaires need to be included, extending the length of the F&D questionnaires. The choice of dietary behaviours is based on dietary challenges among Norwegian adolescents and may vary by country and age groups. Furthermore, the generalizability of future findings may be somewhat limited to semi-urban and highly educated families, such as those living in the south-eastern region and areas surrounding the largest cities in Norway. The pre-test, pilot and test-retest were conducted at one school in a high socio-economic area which might have affected the results to some degree. Despite these limitations, the F&D questionnaires appear to be psychometrically sound tools to assess family characteristics and adolescents’ impulsivity.

## Conclusion

The conceptual framework developed as part of the F&D project may be a useful tool in guiding measurement and assessment of the home food environment and the family processes related to adolescents’ dietary habits. The F&D questionnaires are developed to cover all these aspects, including several important and changeable factors that potentially influence adolescents’ intake of SSB and vegetables. The presented characteristics of the study sample indicate that findings from this study may be applicable to semi-urban and highly educated families. The evaluation of the BIS-Brief in 13–15 year olds and their parents suggests that this scale can be used to assess adolescents’ impulsivity in future studies. The next steps are to explore associations between family processes and the dietary behaviours, bidirectional relationships and gender dyads/triads related to the home food environment. In the long term, it will be interesting to test the questionnaires in assessing family processes longitudinally.

## Additional files

Additional file 1
**BIS-Brief; means (SD), Cronbach’s alpha and intraclass correlation coefficients for the study sample.**
Additional file 2
**Factor loadings (confirmatory factor analysis) for BIS-Brief in the study sample.**
