# Supplementary material for: Development of family and dietary habits questionnaires: the assessment of family processes, dietary habits and adolescents’ impulsiveness in Norwegian adolescents and their parents
Source: Int J Behav Nutr Phys Act. 2014 Oct 15;11:130. doi: 10.1186/s12966-014-0130-z (PMC4200224; doi:10.1186/s12966-014-0130-z)
Supplement: Additional file 1 — BIS-Brief; means (SD), Cronbach’s alpha and intraclass correlation coefficients for the study sample. [file 12966_2014_130_MOESM1_ESM.docx]

Additional file 1: BIS-Brief; means (SD), Cronbach’s alpha and intraclass correlation coefficients for the study sample

|  | **Adolescents** | | | |  | **Mothers** | | | | **Fathers** | | | | **Parents** |
| --- | --- | --- | --- | --- | --- | --- | --- | --- | --- | --- | --- | --- | --- | --- |
|  | **n = 415** | | | | **n = 49** | **n = 238** | | | | **n = 151** | | | | **n = 42** |
| **I /My child…** | **Mean** | **SD** | **CITC** | **α** | **ICC** | **Mean** | **SD** | **CITC** | **α** | **Mean** | **SD** | **CITC** | **α** | **ICC** |
| Total score, impulsivity | 2.00 | (0.49) | - | 0.77 | 0.74 | 1.93 | (0.47) | - | 0.83 | 1.98 | (0.47) | - | 0.82 | 0.77 |
| plan(s) tasks carefully* | 2.27 | (0.84) | 0.39 |  |  | 2.24 | (0.83) | 0.60 |  | 2.38 | (0.80) | 0.61 |  |  |
| do(es) things without  thinking | 2.04 | (0.79) | 0.48 |  |  | 1.89 | (0.63) | 0.55 |  | 1.99 | (0.66) | 0.48 |  |  |
| do(es)n’t “pay attention” | 1.78 | (0.75) | 0.51 |  |  | 1.84 | (0.70) | 0.59 |  | 1.79 | (0.65) | 0.63 |  |  |
| am/is self-controlled* | 1.71 | (0.75) | 0.40 |  |  | 1.71 | (0.70) | 0.62 |  | 1.75 | (0.76) | 0.51 |  |  |
| concentrate(s) easily* | 2.14 | (0.87) | 0.50 |  |  | 1.90 | (0.79) | 0.63 |  | 1.93 | (0.76) | 0.65 |  |  |
| am/is a careful thinker* | 2.09 | (0.79) | 0.58 |  |  | 2.04 | (0.72) | 0.60 |  | 2.09 | (0.80) | 0.59 |  |  |
| say(s) things without  thinking | 1.95 | (0.78) | 0.46 |  |  | 1.79 | (0.63) | 0.39 |  | 1.89 | (0.64) | 0.41 |  |  |
| act(s) on the spur of the moment | 2.01 | (0.77) | 0.45 |  |  | 2.03 | (0.60) | 0.39 |  | 2.03 | (0.62) | 0.42 |  |  |

CITC = Corrected Item-Total Correlation, α = Cronbach’s alpha, ICC: intraclass correlation coefficient,

Rarely/never (1), occasionally (2), often (3) and almost always/always (4), * = reverse coded.
