# Supplementary material for: Development of family and dietary habits questionnaires: the assessment of family processes, dietary habits and adolescents’ impulsiveness in Norwegian adolescents and their parents
Source: Int J Behav Nutr Phys Act. 2014 Oct 15;11:130. doi: 10.1186/s12966-014-0130-z (PMC4200224; doi:10.1186/s12966-014-0130-z)
Supplement: Additional file 2 — Factor loadings (confirmatory factor analysis) for BIS-Brief in the study sample. [file 12966_2014_130_MOESM2_ESM.docx]

Additional file 2: Factor loadings (confirmatory factor analysis) for BIS-Brief in the study sample

|  | **Adolescents** | | **Mothers** | | **Fathers** | |
| --- | --- | --- | --- | --- | --- | --- |
|  | **n = 415** | | **n = 238** | | **n = 151** | |
| Fit indices | p < 0.001, df = 20  RMSEA = 0.166  CFI = 0.731 | | p < 0.001, df = 20  RMSEA = 0.098  CFI = 0.881 | | p < 0.001, df = 20  RMSEA = 0.154  CFI = 0.732 | |
| **I /My child…** | Item loading | Error term | Item loading | Error term | Item loading | Error term |
| plan(s) tasks carefully* | 0.44 | 0.81 | 0.68 | 0.54 | 0.68 | 0.54 |
| do(es) things without thinking | 0.58 | 0.66 | 0.56 | 0.68 | 0.46 | 0.79 |
| do(es)n’t “pay attention” | 0.60 | 0.64 | 0.65 | 0.58 | 0.64 | 0.59 |
| am/is self-controlled* | 0.45 | 0.79 | 0.70 | 0.51 | 0.64 | 0.59 |
| concentrate(s) easily* | 0.57 | 0.67 | 0.74 | 0.45 | 0.79 | 0.38 |
| am/is a careful thinker* | 0.64 | 0.59 | 0.68 | 0.54 | 0.72 | 0.48 |
| say(s) things without thinking | 0.55 | 0.69 | 0.41 | 0.83 | 0.38 | 0.85 |
| act(s) on the spur of the moment | 0.51 | 0.74 | 0.40 | 0.84 | 0.38 | 0.85 |

df = degrees of freedom, RMSEA = Root Mean Square Error of Approximation, CFI = Comparative Fit Index.

Only complete cases were included in the analysis.
